# Antibiotic consumption for sore throat and the potential effect of a vaccine against group A *Streptococcus*: a systematic review and modelling study

**DOI:** 10.1016/j.ebiom.2023.104864

**Published:** 2023-11-09

**Authors:** Kate M. Miller, Timothy C. Barnett, Daniel Cadarette, David E. Bloom, Jonathan R. Carapetis, Jeffrey W. Cannon

**Affiliations:** a Wesfarmers Centre of Vaccines and Infectious Diseases, Telethon Kids Institute, University of Western Australia, Nedlands, Western Australia, Australia; b School of Population and Global Health, University of Western Australia, Nedlands, Western Australia, Australia; c Marshall Centre for Infectious Diseases Research and Training, School of Biomedical Sciences, University of Western Australia, Nedlands, Western Australia, Australia; d Harvard Kennedy School, Harvard University, Cambridge, MA, United States; e Department of Global Health and Population, Harvard T.H. Chan School of Public Health, Harvard University, Boston, MA, United States; f Perth Children’s Hospital, Nedlands, Western Australia, Australia; g Centre for Child Health Research, Medical School, University of Western Australia, Nedlands, Western Australia, Australia

**Keywords:** Group A *Streptococcus*, *Streptococcus pyogenes*, Antibiotics, Sore throat, Tonsillitis, Pharyngitis, Prescription, Acute respiratory infections, Vaccine

## Abstract

**Background:**

Antibiotic consumption can lead to antimicrobial resistance and microbiome imbalance. We sought to estimate global antibiotic consumption for sore throat, and the potential reduction in consumption due to effective vaccination against group A *Streptococcus* (Strep A).

**Methods:**

We reviewed and analysed articles published between January 2000 and February 2022, identified though Clarivate Analytics’ Web of Science search platform, with reference to antibiotic prescribing or consumption, sore throat, pharyngitis, or tonsillitis. We then used those analyses, combined with assumptions for the effectiveness, duration of protection, and coverage of a vaccine, to calculate the estimated reduction in antibiotic prescribing due to the introduction of Strep A vaccines.

**Findings:**

We identified 101 studies covering 38 countries. The mean prescribing rate for sore throat was approximately 5 courses per 100 population per year, accounting for approximately 5% of all antibiotic consumption. Based on 2020 population estimates for countries with empiric prescribing rates, antibiotic consumption for sore throat was estimated to exceed 37 million courses annually, of which half could be attributable to treatment for Strep A. A vaccine that reduces rates of Strep A infection by 80%, with 80% coverage and 10 year’s duration of protection, could avert 2.8 million courses of antibiotics prescribed for sore throat treatment among 5-14 year-olds in countries with observed prescribing rates, increasing to an estimated 7.5 million averted if an effective vaccination program also reduced precautionary prescribing.

**Interpretation:**

A vaccine that prevents Strep A throat infections in children may reduce antibiotic prescribing for sore throat by 32–87% depending on changes to prescribing and consumption behaviours.

**Funding:**

The Wellcome Trust, grant agreement number 215490/Z/19/Z.

**Research in context**

**Evidence before this study**

We conducted a global systematic review of the worldwide literature to identify data on the rate of antibiotic prescribing or consumption to treat sore throat among the general population, the proportion with known aetiology of group A *Streptococcus* (Strep A), and the distribution of antibiotic classes prescribed for sore throat. We used Clarivate Analytics’ Web of Science (WoS) search platform, which includes the WoS Core Collection and Medline, to search for articles published between January 2000 and February 2022. Search terms included tonsillopharyngitis, pharyngitis, sore throat, throat infection, and antibiotics. No restrictions on language were applied.

**Added value of this study**

To our knowledge, this is the first study to compile worldwide data from peer-reviewed and grey literature on the rate, and class, of antibiotics prescribed to treat sore throat and the proportion of prescribed antibiotic courses that can be attributed to Strep A. There was limited data from low- and middle-income countries. We used available data to model the potential impact of Strep A vaccines based on assumptions for their direct impact on infection rates and their potential impact on precautionary prescribing practices. Our analyses suggest that the latter assumption has as much, if not more, importance on overall reductions in antibiotic prescribing as differences in vaccine efficacy, coverage, and duration of protection.

**Implications of all the available evidence**

Our study found limited empirical data to estimate the global consumption of antibiotics due to sore throat, and more specifically for Strep A infection. These data are crucial to understanding the broader impact of Strep A vaccination—impact beyond a direct reduction in infections–and, therefore, its wider economic and societal value.

## Introduction

Infection by *Streptococcus* pyogenes, also known as group A *Streptococcus* (Strep A), is the most common bacterial case of sore throat (e.g., pharyngitis or tonsillitis), collectively causing more than 600 million cases per year globally.^1^ These infections may lead to several severe clinical sequelae such as streptococcal toxic shock syndrome, sepsis, necrotising fasciitis, acute rheumatic fever (ARF) and subsequent rheumatic heart disease, and acute post-streptococcal glomerulonephritis.

Accurate diagnosis and antibiotic treatment of Strep A sore throat is known to reduce the risk of developing ARF and may reduce the risk of other severe sequelae.^2^ However, while Strep A is the most common bacterial cause of acute sore throat, most sore throats are caused by viral infections where antibiotics are ineffective.^2^ Difficulties in clinically discriminating between pharyngitis due to Strep A and other pathogens, along with the time and cost associated with performing and processing a diagnostic test, means that many patients are prescribed antibiotics either without testing or before the test result is known.^3^ In low-resource settings, such tests are often not available, practical, or affordable.

The frequent occurrence of sore throat infections combined with difficulties in discriminating Strep A infections from viral aetiology results in sore throat being one of the most common reasons for antibiotic prescription globally. For example, analysis of the most recent Eurobar data on antimicrobial resistance (AMR) shows that sore throat was the second most common reason for antibiotic prescription in Europe, responsible for 14% of all antibiotics consumed by adults.^4^ Additionally, a US study found that sore throat (pharyngitis) was the third most frequent reason for antibiotic prescribing.^5^

Inappropriate and excessive antibiotic consumption is a major contributor to AMR.^6^^,^^7^ AMR reduces antibiotic effectiveness, worsens patient outcomes, and increases treatment costs.^8^ Immunization with a future Strep A vaccine can prevent necessary prescription of antibiotics for sore throat by preventing Strep A infection, and it will likely reduce unnecessary prescription of antibiotics for sore throat by reducing the probability that any given case is caused by Strep A and therefore reducing antibiotic prescribing for suspected Strep A pharyngitis. As such, the development of a Strep A vaccine has been proposed by the World Health Organisation (WHO) as a global priority for reducing antibiotic consumption and stemming AMR.^9^^,^^10^

Understanding the full scope of antibiotic consumption for sore throat is a key part of understanding the potential value of Strep A vaccination. Therefore, for countries with observational data, this study aimed to: (1) estimate the mean rate and total number of antibiotic courses prescribed to treat sore throat; (2) estimate the proportion of prescriptions for sore throat that is attributable to treatment of sore throat caused by Strep A; (3) summarise the distribution of antibiotic classes prescribed for sore throat, and (4) explore the potential reduction in antibiotic prescribing for sore throat due to implementation of prospective Strep A vaccines.

## Methods

We conducted a systematic review to identify, collate, and analyse data related to aims one through three. We then used those analyses, combined with assumptions for the effectiveness, duration of protection, and coverage of a vaccine, to calculate the estimated reduction in antibiotic prescribing due to the introduction of Strep A vaccines.

### Search strategy and selection criteria

We conducted a systematic review for aims 1–3 in accordance with PRISMA guidelines.^11^ We used Clarivate Analytics’ Web of Science (WoS) search platform, which includes multiple databases such as the WoS Core Collection, Medline, Data Citation Index, KCI-Korean Journal Database, Russian Science Citation Index, and the SciELO Citation Index, to search for articles published between January 2000 and February 2022. Our search used the following Topic Search terms: (tonsillopharyngitis OR pharyngitis OR sore throat OR throat infection) AND (antibiotic OR antimicrobial OR antibact∗ OR prescri∗) NOT (postoperat∗ OR post-operat∗). We supplemented database searches by searching Google Scholar for grey literature, and manually reviewing reference lists of eligible studies identified during the search. No restrictions on the language were applied. The search strategy was conducted by JWC.

Studies were considered for inclusion in one or more of our analyses if they evaluated one of three outcomes of interest: (1) rate of antibiotic prescription for sore throat among the general population, (2) distribution of antibiotic prescription for sore throat by laboratory or point-of-care diagnosis, or (3) distribution of antibiotic prescriptions for sore throat by antibiotic class. We included observational studies and control arms of randomized control trials of treatment regimens. We restricted studies to those among primary care patients, but we included studies conducted in primary care settings that also involved outpatient and emergency departments. We excluded studies conducted solely in hospital-based outpatient or emergency departments as treatment of sore throat in those settings is relatively rare compared to primary care and likely reflect prescribing rates among severe cases. Studies of attitudes towards antibiotic consumption with no primary data on prescribing or consumption were also excluded, as were studies in which participants who were surveyed on antibiotic consumption that were recruited from pharmacies, to limit biases towards treatment. Studies on post-operative sore throat were considered inappropriate for this review and were excluded. Search results were uploaded to Endnote X9 for deduplication and cataloguing. Two authors (JWC and KM) independently screened and reviewed the titles and abstracts of potential studies against the eligibility criteria. Papers meeting the inclusion criteria were sourced in full text for final review for inclusion. Google Translate was used to translate non-English language papers; however, the search was conducted in English. Differences in opinion between the two reviewers were resolved by discussion to achieve consensus.

#### Data extraction

Following full text review, two reviewers (JWC, KM) independently extracted data from each of the eligible studies using a standardised proforma. Variables included: authors; year of publication; study period; study location (i.e., country, city, or region); study setting; number of participants; participant age range, sex, and population group; diagnosis (case definition, diagnostic methods and results); and outcome measures (number of cases of sore throat, number of cases for which antibiotics were prescribed or consumed, number of confirmed Strep A infections, and antibiotic class).

#### Case definitions

The primary outcome tracked (or “monitored”) was sore throat, including pharyngitis and tonsillitis. A secondary outcome tracked was sore throat caused by Strep A. A case of sore throat was defined as any illness in a participant who reported symptoms consistent with a sore throat or who was clinically diagnosed with pharyngitis or tonsillitis, regardless of other clinical symptoms. A case of Strep A sore throat was defined as illness in a participant who complained of sore throat or who had clinical signs of pharyngitis or tonsillitis combined with microbiologic confirmation of Strep A in the oropharynx by a positive throat culture, rapid antigen detection test (RADT), nucleic acid amplification test, or another appropriate molecular test.

The appropriateness of antibiotic prescribing was assessed based on adherence to recommended antibiotics as listed in current country or regional specific guidelines. Antibiotic classes were categorised according to the Anatomical Therapeutic Chemical Classification System, controlled by the WHO Collaborating Centre for Drug Statistics Methodology.^12^

We defined outcomes to be among children and young adults when study participants were predominately <20 years old and among adults when participants were predominately ≥20 years old. However, we estimated the numbers of antibiotics prescribed and potentially averted by vaccination for children aged 5–14 years as that age range aligned with our assumed vaccination parameters (i.e., a vaccine given at age five and has an expected duration of protection of 10 years; see below).

#### Quality assessment

Articles eligible for inclusion in our meta-analysis for the proportion of prescriptions attributable to Strep A were independently assessed for quality by two reviewers (KM and JWC) using the Joanna Briggs Institute (JBI) “*Checklist for Prevalence Studies*”.^13^ The checklist is composed of nine questions that related to study selection, measurement, and comparability of studies that the reviewers considered for each study, thus scores could range from zero to maximum nine. Differences in scoring were resolved through discussion.

### Data summary and analysis

All statistical analyses and plotting were performed using R (version 4.1.0) statistical software.^14^ Our study was registered with PROSPERO, number: CRD:42021212544.

#### Rates and numbers of antibiotic courses prescribed to treat sore throat

We summarised the rates of antibiotic prescribing for sore throat by study country and by age group at the time of treatment. Prescribing rates were defined as the number of courses to treat sore throat per 100 population per year, which were acquired directly from the study or calculated using the data reported by the study. Some studies reported prescribing rates stratified by several age groups, in which case we combined age-specific rates to match our definitions of children and young adults and adults as closely as possible. Demographic data from the United Nations World Population Prospects were used for nationally representative data (i.e., estimates for the number of prescriptions for sore throat at a national level) when population denominators were not reported.^15^

Using the country- and age-specific prescribing rates, we calculated the arithmetic mean prescribing rate and the population-weighted mean prescribing rate of all countries for each age group. For countries with more than one study among a particular age-group, we used the most recent or nationally representative prescribing rate because most within-country studies were updated analyses of the same or similar data sources.

We also used the most recent or nationally representative prescribing rate to estimate the number of antibiotic courses prescribed to treat sore throat in 2020 among each country analysed in our review. Here, we estimate the number of courses prescribed to treat sore throat among children aged 5–14 years and among the total population (all ages) by multiplying country-specific prescribing rates for children and young adults and for all ages by, respectively, the estimated 2020 population aged 5–14 years and total population.

Additionally, we estimated the percent of total antibiotic prescription for any health conditions or infections that was due to sore throat treatment for each county. We estimated the number of defined daily doses (DDDs) per 1000 inhabitants per day (DID) using the method described elsewhere^16^ and assuming an average antibiotics course to treat sore throat comprised 10 g of amoxicillin (i.e., 500 mg twice per day for 10 days). For each country, we compared our estimated DID to treat sore throat to the total DIDs for all antibiotics reported by the WHO.^16^ Where data was unavailable for a specific country, we used the estimated average DIDs consumed globally in 2015 by Klein et al.^17^

#### Courses attributable to Strep A

We conducted a random-effects meta-analysis of the proportion of all prescriptions for sore throat that were diagnostically confirmed as Strep A sore throat (i.e., for each study, the number of prescriptions among patients diagnostically confirmed to have Strep A divided by the total number of prescriptions, regardless of whether all prescriptions were linked to a test result). We conducted sub-group meta-analyses by age group (children and young adults and adults) at time of treatment and by study country. Between-study heterogeneity was assessed using the I^2^ and Cochran’s Q tests.

#### Effect of Strep A vaccination

An effective Strep A vaccination strategy would reduce the incidence of Strep A infection and may influence antibiotic prescribing practices for sore throat. Over the last decade, the benefit of antibiotic treatment for sore throat of any aetiology in populations where ARF, rheumatic heart disease, and other severe complications of Strep A pharyngitis are rare has been debated.^18^ Therefore, we estimated the potential number of prescriptions that could be averted due to Strep A vaccination under two scenarios.

Scenario 1 (no change in prescribing practices) assumed that there would be no change in prescribing practices but that the proportion of prescriptions for sore throat that are attributable to Strep A (extrapolated from our meta-analysis) would be averted by vaccination; the modelled effect of vaccination against Strep A infection is described at the end of this section. While an effective vaccine would prevent infection among cases that may not traditionally be prescribed antibiotics, our analysis aimed to explore only the reductions in antibiotic prescriptions rates and not in both treated and untreated infection rates.

Scenario 2 (reduced prescribing rates in HICs) assumed that there would be a change in prescribing practices among children in HICs presenting with sore throat in conjunction with a decrease in Strep A infection rates. In HICs, prescribing rates were assumed to match, based on this review, the country with the lowest prescribing rate among children, while in low- and middle-income countries (LMICs), prescribing practices were assumed to be unchanged. Given the revised (i.e., lower) antibiotic prescribing rates, we then assumed, as in Scenario 1, that a proportion of prescriptions attributable to Strep A would be averted by vaccination.

In both scenarios, the proportion of prescriptions attributable to Strep A that could be prevented by vaccination were based on the following assumptions: vaccination at age five years; vaccine effectiveness (for prevention of Strep A pharyngitis) of 80%, based on the WHO’s Preferred Product Characteristics for Strep A vaccines^19^; vaccine coverage at 80% of the five-year-old population; and vaccine duration of protection of 10 years with no waning during that period.

Additionally, we conducted sensitivity analyses to explore the impact of uncertainty in vaccine coverage (70–90%), efficacy against Strep A pharyngitis (70–90%), and duration of protection (5 and 10 years) on the potential numbers of antibiotics averted by Strep A vaccination. The effect of a reduced duration of protection to 5 years was mirrored in a reduced age-range for changes in prescribing practices under Scenario 2; the minimum rate was applied only to 5-9 year-olds, while prescribing among 10-14 year-olds was modelled as observed. Further details for these calculations are provided in the Supplementary Material.

#### Antibiotic class prescribed

Descriptive statistics were used to describe the distribution of prescriptions by antibiotic class and calculate the rate of appropriate antibiotic prescriptions. To estimate levels of appropriate antibiotic prescribing, we compared antibiotics prescribed with the antibiotics recommended in relevant national or regional treatment guidelines. Data was excluded for studies conducted in countries where guidelines were not available. We calculated the average distribution of classes prescribed across studies with equal weighting.

### Role of the funding source

The funder of the study had no role in the study design, data collection, data analysis, data interpretation, or writing of the report. Authors were not precluded from accessing data in the study, and all authors had final responsibility for the decision to submit for publication.

## Results

### Search results (all outcomes combined)

**Fig. 1:**
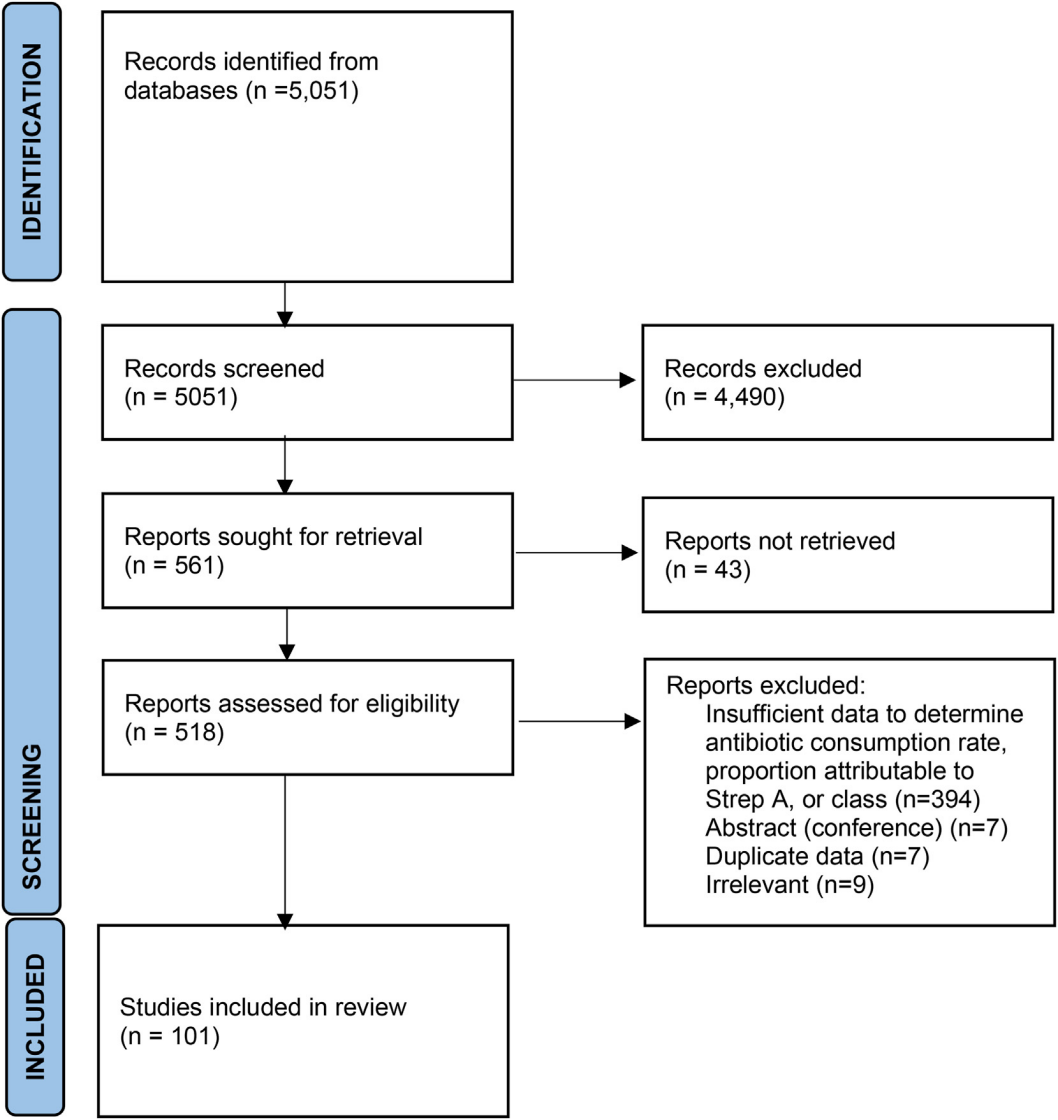
**PRISMA 2020 flow diagram for new systematic reviews which included searches of databases and registers only**.^139^

We identified 5051 studies, of which 561 studies were retained for full text review after title and abstract screening. Subsequently, 101 studies covering 38 countries were included in one or more analyses (Fig. 1; Supplementary Table S1).

### Antibiotic prescribing for sore throat

We identified 44 studies from 19 countries reporting prescribing rates; one study covered three countries and five studies comprised rates stratified by multiple age groups (i.e., children, adults, and all ages combined). Twenty-seven studies from 12 countries included rates for all ages (Supplementary Table S2),^5^^,^^20^, ^21^, ^22^, ^23^, ^24^, ^25^, ^26^, ^27^, ^28^, ^29^, ^30^, ^31^, ^32^, ^33^, ^34^, ^35^, ^36^, ^37^, ^38^, ^39^, ^40^, ^41^, ^42^, ^43^, ^44^, ^45^ 16 studies from 11 countries included rates for children and young adults (Supplementary Table S3),^5^^,^^21^^,^^31^^,^^35^^,^^36^^,^^46^, ^47^, ^48^, ^49^, ^50^, ^51^, ^52^, ^53^, ^54^, ^55^, ^56^ and 11 studies from six countries included rates for adults (Supplementary Table S4).^5^^,^^21^^,^^31^^,^^35^^,^^36^^,^^57^, ^58^, ^59^, ^60^, ^61^, ^62^ Studies were predominantly from the US (*n* = 11), UK (*n* = 7), the Netherlands (*n* = 6), and Sweden (*n* = 5). Studies from low- and middle-income countries were underrepresented (n = 3; Thailand,^28^ Serbia,^47^ and Zambia,^55^). The data collection period across these studies covered 31 years from 1987 to 2017. The studies ranged in their inclusion of settings within primary care. The most recent study from the US comprised general practitioners (GPs), outpatient and emergency departments, and retail clinics.^35^ Most studies reported antibiotic prescribing data, but eight (18%) studies reported dispensing data.^20^^,^^23^^,^^24^^,^^36^^,^^45^^,^^50^^,^^53^^,^^55^

**Fig. 2:**
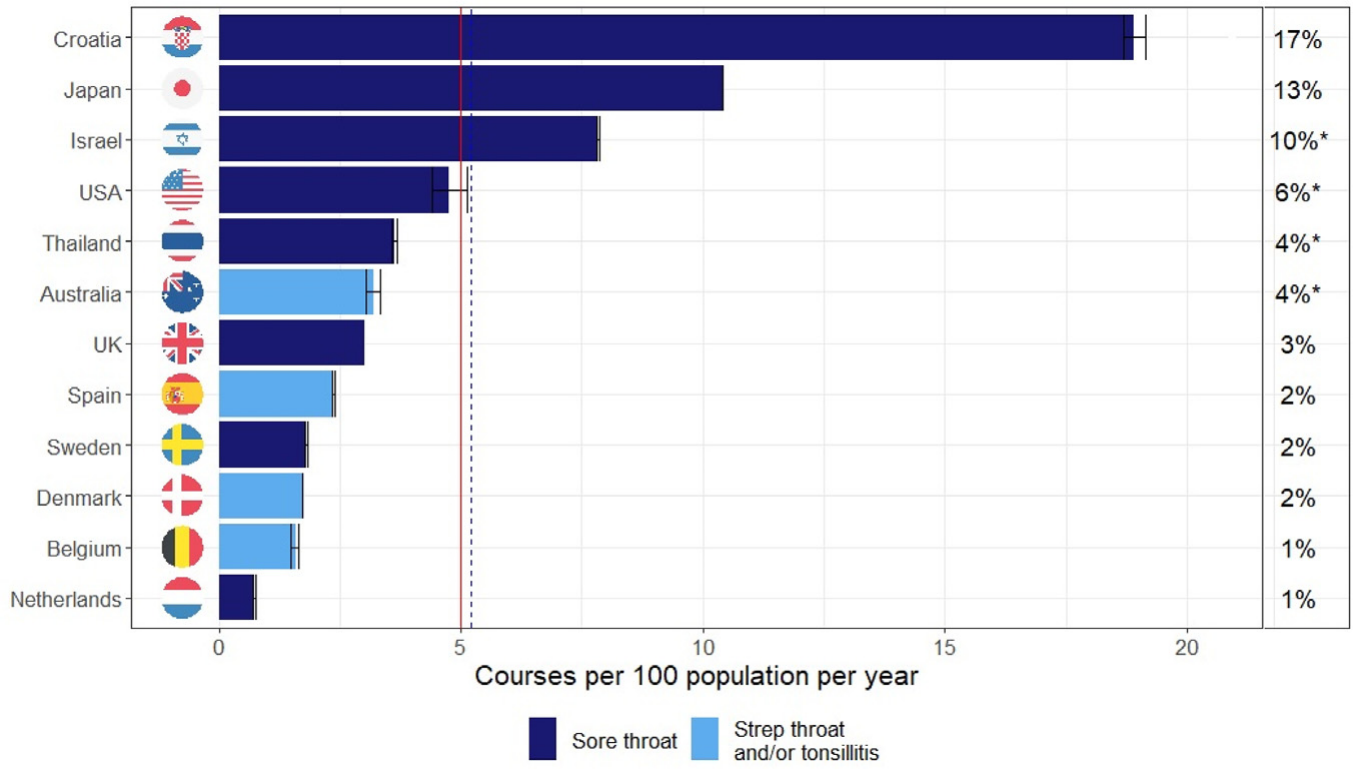
**Antibiotic prescribing rate (bars) and percent of all antibiotic consumption that is due to prescriptions for sore throat (figures on the right) by country, all ages.** Sore throat comprises “sore throat” or pharyngitis with or without tonsilitis. Population-weighted mean (BLUE dashed) = 5.2; Arithmetic mean (RED) = 5.0 (right) Estimated % of all antibiotic consumption measured in Defined Daily Doses. ∗Relative to global mean in the absence of country-specific consumption.

Based on data from the most recent year(s) or nationally representative studies among all age groups (n = 12), the mean and population-weighted mean rates of antibiotic courses prescribed for sore throat were 5.0 and 5.2 per 100 population per year, respectively (Fig. 2). At a country level, prescriptions for treatment of sore throat ranged between an estimated 1–17% of all antibiotics prescribed; the average across countries was 5.5% of all antibiotic prescriptions.

**Fig. 3:**
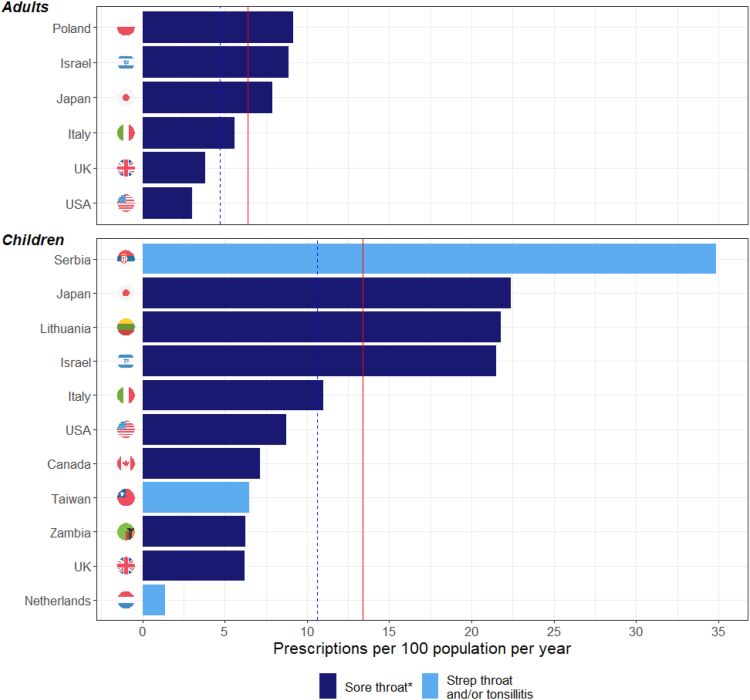
**Antibiotic prescribing rate by age group ∗Sore throat comprises “sore throat” or pharyngitis with or without tonsilitis.** Children–Population-weighted mean (BLUE dashed) = 10.7; Arithmetic mean (RED) = 13.4. Adults–Population-weighted mean (BLUE dashed) = 4.8; Arithmetic mean (RED) = 6.4.

Among studies with rates for children and young adults (including four of five all-age studies with age-stratified rates), the mean and population-weighted mean rates of antibiotic courses prescribed for sore throat were 13.4 and 10.7 per 100 population per year, respectively (Fig. 3). For adults, rates were 6.4 and 4.8 per 100 population per year, respectively (Fig. 3). The mean rates of antibiotic prescribing for children and adults based on age-specific data were both higher than the mean rate for all-ages, which is a consequence of the age-specific and all-age means being derived from different populations.

For 2020, we estimate that 8.6 million antibiotic courses were prescribed for sore throat among children aged 5–14 years (8.0 million among the HICs reviewed) and 37.4 million antibiotic courses for all ages.

### Prescriptions attributable to Strep A

Nineteen studies across nine countries reported prescriptions for diagnostically confirmed Strep A pharyngitis (Supplementary Table S5).^26^^,^^40^^,^^63^, ^64^, ^65^, ^66^, ^67^, ^68^, ^69^, ^70^, ^71^, ^72^, ^73^, ^74^, ^75^, ^76^, ^77^, ^78^ All studies were conducted in HICs, of which seven were in the US, four in Sweden, two in Spain, and the remainder in other countries. Seven studies reported diagnostic results among all age groups (but one study did not report counts), six studies among only children and five studies among only adults.

**Fig. 4:**
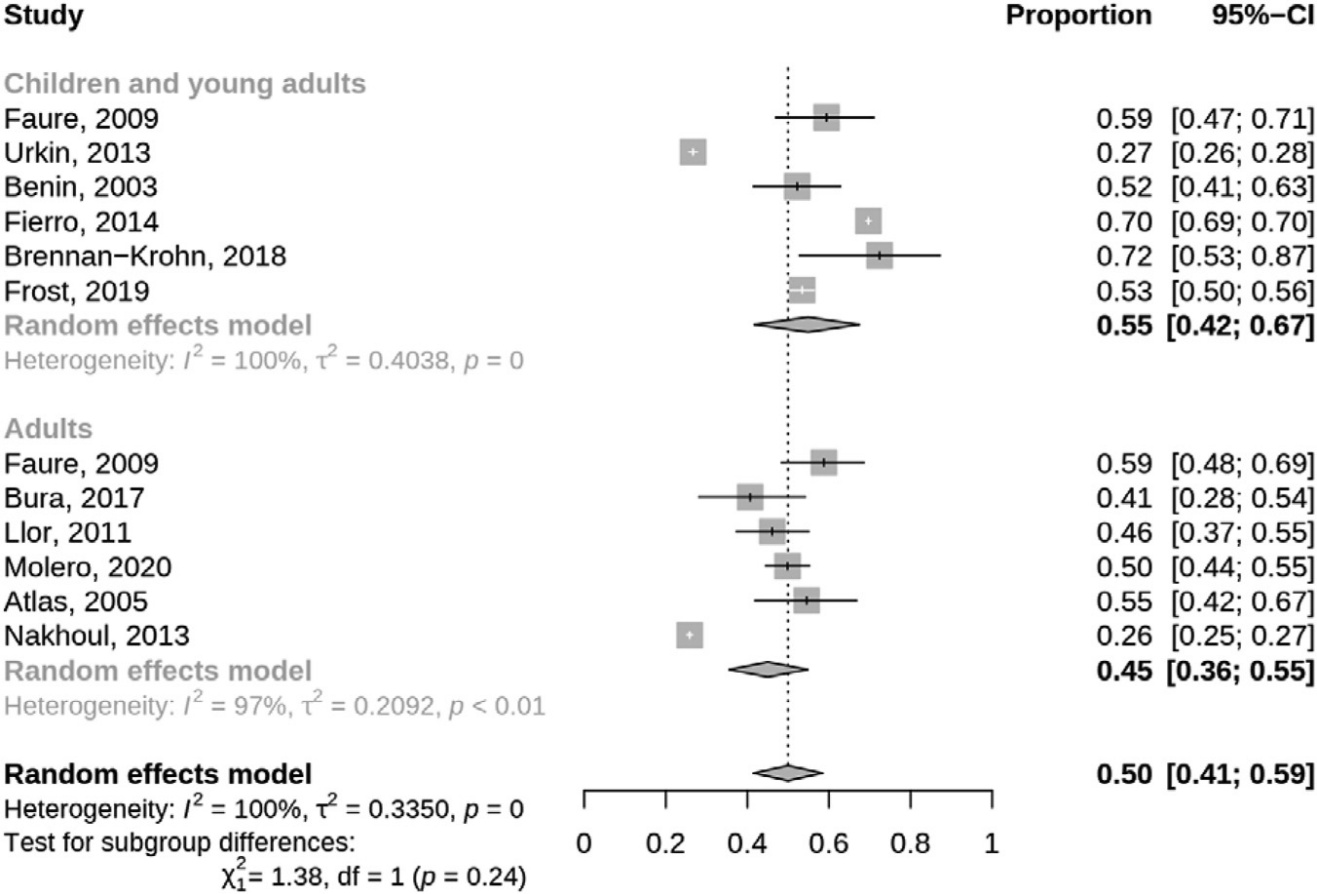
**Random-effects meta-analyses for proportion of sore throat prescriptions attributable to Strep A by age group; children and young adults predominately aged < 19 years**.

Of all antibiotic prescriptions for sore throat, 50% (95% CI 41–59%) were for Strep A positive patients, and the remaining proportion were for Strep A negative patients or those not tested (the proportions of patients prescribed antibiotics that were Strep A positive and that were not tested for each study are shown in Supplementary Table S5). There was no statistical difference between children and young adults and adults in the pooled proportion of patients who were prescribed antibiotics for sore throat that were Strep A positive (p = 0.24, Fig. 4). There was a difference between study country (p < 0.001; Supplementary Fig. S1), where the single study from Israel had a lower proportion of prescriptions among Strep A positive patients compared to the pooled proportion for the US (p = 0.022). However, there was significant statistical heterogeneity between the US studies (p < 0.001).

Using the JBI’s critical appraisal checklist tool for prevalence studies to access the quality of studies included in our meta-analysis of prescriptions attributable to Strep A, one study attained a score of nine,^40^ four studies attained a score of eight,^65^^,^^70^^,^^73^^,^^75^ two studies attained a score of seven,^71^^,^^78^ five studies attained a score of six,^26^^,^^64^^,^^66^^,^^69^^,^^72^ and six studies attained a score of five or less (Supplementary Table S6).^67^^,^^68^^,^^74^^,^^76^^,^^77^ Minimal risk of bias was observed for study sampling and validity of measurements for confirming cases of Strep A sore throat. The main sources of potential bias were related to the assessment of coverage bias and study comparability due to insufficient detail on study participants and setting.

### Global estimated prescription of antibiotics for sore throat and impact of a Strep A vaccines

Using the UN’s 2020 population estimates, the countries included in our analyses represent 9% of the global population for all age groups (47% of HIC populations) and 6% of the global population aged 5–14 years (53% of children from HICs and 1% of children from middle income countries). There were no studies from low-income countries.

Based on our meta-analyses, we assumed that 50% of all prescriptions for sore throat are due to Strep A and that this estimate did not differ by age group or country; the latter assumption was due to the limited number of studies from each country and the significant statistical heterogeneity among studies within the US. Thus, estimates for the potential reduction in antibiotic courses prescribed to treat sore through due to vaccination are shown in Table 1. Under the assumption that vaccination will prevent Strep A infections but not effect prescribing practices (Scenario 1), 2.8 million antibiotic courses may be averted among children, a 32% reduction among children and 7% reduction among the whole population. The reduction in prescribing could increase to 7.5 million antibiotic prescriptions averted (87% of all antibiotic prescriptions among children for Strep A sore throat) if, as described in Scenario 2, prescribing practices were reduced in HIC to rates matching the Netherlands (1.4 courses per 100 population per year) and Strep A infection rates were reduced among a proportion of vaccinated children who may have otherwise had a prescription attributable to Strep A.

**Table 1:** Estimated 2020 reductions in antibiotic courses prescribed to treat pharyngitis among children (5-14 years-old) under two scenarios of the impact of global Strep A vaccine implementation.

Scenario	Assumptions	Prescriptions averted (percent of prescriptions averted among total antibiotic courses for pharyngitis)
1 No change in prescribing practices (minimum estimate of averted prescriptions)	Vaccination averts a proportion of sore throat prescriptions attributable to Strep A infection	**2.8 million (32%)**
2 Change in prescribing practices in HICs; no change in LMICs	In HICs^a^	7.3 million (91%)
	In LMICs^b^	0.2 million (32%)
	Total	**7.5 million (87%)**

Bolded figures represent the total prescriptions averted for each scenario.

a In high-income countries (HICs), prescribing practices for sore throat will be reduced to match the HIC with the lowest observed prescribing rate among children (the Netherlands; 1.4 courses per 100 persons per year) and, subsequently, a proportion of sore throat prescriptions that are attributable to Strep A infection will be averted by vaccination.

b In low- and middle-income countries (LMICs), only a proportion of sore throat prescriptions that are attributable to Strep A infection will be averted by vaccination.

**Fig. 5:**
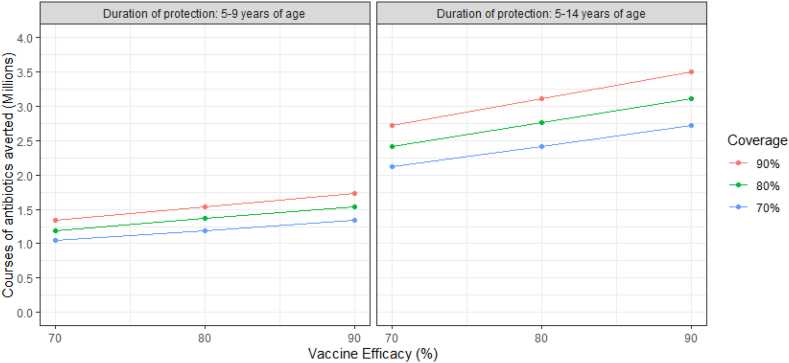
**Sensitivity analysis for the reduction in antibiotic prescribing for sore throat due to Strep A vaccination (Scenario 1: No change in prescribing practices; vaccination averts a proportion of sore throat prescriptions attributable to Strep A infection)**.

The sensitivity analyses indicate that, with lower vaccine efficacy (70%) and coverage (70%), more than 2.1 million antibiotic courses may be averted among children under Scenario 1 (24.5% reduction in prescribed courses), increasing to approximately 3.5 million courses averted (40.5% reduction) with higher vaccine efficacy (90%) and coverage (90%; Fig. 5). The corresponding results for a vaccine with five years’ duration of protection are 1.0 million and 1.7 million antibiotic courses averted, equating to a 12.1% and 20.0% reduction in antibiotic prescribing to treat sore throat among children.

For the sensitivity analyses of Scenario 2, the difference in averted courses for changes in vaccine efficacy, coverage, and duration of protection, are outweighed by the impact from the assumed change in prescribing practices. In this scenario, over 7 million and over 3 million antibiotic courses are averted for vaccines with durations of 10 years and 5 years, respectively (Supplementary Fig. S2). For vaccines with a 10-year duration of protection, 85.3% and 88.4% of antibiotic courses are averted among children due to vaccines with 70% efficacy and coverage and 90% efficacy and coverage, respectively. The corresponding figures for vaccines with a 5-year duration of protection were 42.1% and 43.6%.

### Antibiotic class of prescriptions for sore throat

Data on antibiotic class were available in 62 studies, reporting on 52.3 million prescriptions for inclusion in analysis. Nineteen studies on prescribing practices for sore throat were of children,^46^^,^^50^^,^^69^^,^^70^^,^^79^, ^80^, ^81^, ^82^, ^83^, ^84^, ^85^, ^86^, ^87^, ^88^, ^89^, ^90^, ^91^, ^92^, ^93^, ^94^ eight studies were of adults,^57^, ^58^, ^59^^,^^61^^,^^62^^,^^95^, ^96^, ^97^ 34 studies covered all age groups,^20^^,^^24^^,^^25^^,^^28^^,^^31^^,^^34^^,^^37^^,^^40^^,^^45^^,^^63^^,^^72^^,^^77^^,^^98^, ^99^, ^100^, ^101^, ^102^, ^103^, ^104^, ^105^, ^106^, ^107^, ^108^, ^109^, ^110^, ^111^, ^112^, ^113^, ^114^, ^115^, ^116^, ^117^, ^118^, ^119^ and one study did not state participants ages.^92^ Most studies were from HICs (*n* = 50; 82%), seven were from four upper-middle income countries (Turkey, Thailand, Brazil, and Bosnia & Herzegovina), and four were from three lower-middle income countries, (Indonesia, India, and Pakistan). No studies from low-income countries were available for inclusion. The most commonly prescribed antibiotics for sore throat across studies were ‘penicillin beta-lactam antibacterials’ (JO1C) (70.1%), of which penicillins with extended spectrum (J01CA; e.g., amoxicillin) were most commonly prescribed (Supplementary Table S7). The second most common antibiotic class was ‘macrolides, lincosamides and streptogramins’ (JO1F) (12.6%), which were almost exclusively macrolides (J01FA). ‘Other beta-lactam antibacterials’ (JO1D) (11.0%) made up most of the remaining prescriptions. The distribution of the different generation (1st, 2nd, and 3rd) cephalosporins was relatively even (35%, 39%, and 26%, respectively). No notable differences were observed between the antibiotic classes prescribed for children and adults with sore throat. The study size and distribution of antibiotic classes prescribed between country income level varied considerably (Supplementary Fig. S3).

Data by individual type of antibiotic were provided in 57 of the 62 studies. The portion of patients prescribed a guideline-recommended antibiotic ranged from 0 to 100%; median 69%. Combinations of penicillins and beta-lactamase inhibitors and macrolides were the most common antibiotic classes prescribed against guideline recommendations, accounting for 39.2% and 29.7% of non-recommended antibiotics prescribed respectively. Amoxicillin-clavulanate was the most used non-recommended antibiotic.

## Discussion

Our objectives were to summarise antibiotic prescriptions for sore throat by reviewing and synthesizing data from the literature and, subsequently, to explore the potential effect of a Strep A vaccine on global antibiotic consumption. However, almost all studies on antibiotic consumption for sore throat were from HICs. From the studies included for analysis, we calculated a weighted mean prescribing rate for sore throat of approximately five courses per 100 persons per year among all age groups and 13.4 courses among children and young adults. We also calculated that, among prescriptions linked to diagnostic testing results, half of all prescriptions for sore throat were given for infections caused by Strep A; the remainder were either negative for Strep A or not tested.

From our findings, we estimate that, in 2020, 37.4 million and 8.6 million courses of antibiotics were prescribed population wide (all ages) and to children aged 5–14 years in the reviewed countries. The implementation of a Strep A vaccine, if administered to 5-year-old children, could avert over 2.7 million courses of antibiotics prescribed for Strep A pharyngitis by directly preventing infection, which is a 32% reduction in prescriptions among 5-14 year-olds. If, in addition to directly preventing Strep A infection, vaccination reduces the rate of precautionary prescribing in HICs, then we estimate that almost 7.5 million courses of antibiotics prescribed for sore throat among children could be averted. As indicated by the results of our sensitivity analyses, the assumed impact of a vaccine on prescribing practices has as much, if not more, importance as differences in vaccine efficacy, coverage, and duration of protection. Under Scenario 1 (no changes in prescribing practices), the relative difference in averted prescriptions increased 1.65 fold when vaccine efficacy and coverage increased from 70% to 90%; this relative difference was the same for either duration of protection assumption. The relative difference in averted prescribing under Scenario 2 (changes in prescribing practices among HICs) was just a 1.04 fold increase when vaccine efficacy and coverage rates increased. Further research could explore the impact that those vaccination parameters have on effecting changes in prescribing practices.

Our estimated reductions in antibiotic prescribing to treat sore throat do not include the potential reduction in Strep A sore throat due to herd protection among non-vaccinated children and adults, nor does it account for the potential to alleviate the need for antibiotic use for sore throat globally.^35^^,^^120^

Lewnard et al.^35^ recently estimated that a vaccination program (assuming the WHO target of 80% efficacy) would prevent 3.8% of antibiotic prescriptions for pharyngitis annually among US children aged 3–9 years who are vaccinated during infancy and again at 4 years of age. The estimated reduction was lower than our primary estimate due, likely, to the differences in study populations and key methodological differences. First, the US study estimated the proportion of prescriptions attributable to Strep A by (a) estimating the proportion of pharyngitis patients that were due to Strep A infection based on a meta-analysis of studies reporting pathology data among pharyngitis patients and then (b) attributing observed prescriptions among pharyngitis patients between predicted Strep A and non-Strep A pharyngitis patients from (a). In our study, we estimated the proportion of prescriptions attributable to Strep A directly by conducting a meta-analysis of studies reporting pathology data among the subset of pharyngitis patients that were prescribed antibiotics. We believe our approach better accounts for the signs and symptoms suggestive of Strep A pharyngitis that likely results in a higher proportion of prescriptions among patients with a Strep A infection compared to those with a viral infection. Second, the study by Lewnard et al.^35^ modelled exponential waning of vaccine duration of protection, whereas we assumed the vaccine would remain at full efficacy for ten years. Either assumption is plausible as the duration of acquired immunity from Strep A infection is not well understood and none of Strep A vaccine candidates have undergone efficacy trails, let alone post-licensure analyses of effectiveness in the general population. Differences in vaccine duration of protection assumption demonstrate an important area for further consideration as vaccine development progresses.

To our knowledge, we present the first meta-analysis for the proportion of antibiotic prescriptions to treat sore throat that are attributable to Strep A based on diagnostic outcomes among patients receiving an antibiotic prescription. Our results, which indicated that 50% of all prescriptions for sore throat were microbiologic test-confirmed Strep A infections, suggest that there is room for improved antimicrobial stewardship. It is possible that some proportion of prescriptions that were for sore throat infections not due to Strep A were not dispensed or consumed, particularly in populations practicing delayed consumption pending testing results. However, a proportion (ranging from 5% to 51%; Supplementary Table S5) of patients prescribed antibiotics in several studies were not tested at all, and one study reported that 84% of prescribed antibiotics were dispensed to the patient.^36^ Studies that have investigated the reasons for inappropriate antibiotic prescribing have noted meeting perceived and real patient expectations and pressure, a desire to maintain positive relations with patients, diagnostic uncertainty, limited access to diagnostic testing, and organizational culture as key drivers for antibiotic prescribing.^36^^,^^121^ Ultimately, antibiotic prescribing to patients who are unlikely to benefit is not benign. All antibiotic prescribing has the potential to be consumed and impact the prevalence of antibiotic-resistant bacteria.^57^

One of our major findings was the lack of studies from low- and middle-income countries, where rates of Strep A disease, including severe complications of pharyngitis such as invasive disease and rheumatic heart disease, are much higher than in HICs.^1^ In these countries, the availability of clinicians and Strep A diagnostic tests are limited and unaffordable and antibiotic use is often less regulated, with widespread over the counter dispensing.^122^ Understanding sore throat-based antimicrobial use in these countries is critical and may be a major driver of AMR. Equally, the impact of a Strep A vaccine may be even more dramatic in such settings.

There is concern that broad spectrum antibiotics are being unnecessarily prescribed to people with sore throat. There are two main concerns with the use of these agents. The first is cost and the second is the development of bacterial antibiotic resistance.^61^ Antibiotic treatment guidelines for Strep A pharyngitis vary by country and by patient risk for severe Strep A diseases such as acute rheumatic fever.^120^^,^^123^^,^^124^ Criteria for antibiotic therapy include various combinations of symptomatic diagnostic criteria (e.g., modified Centor score) and/or confirmation of Strep A infection using rapid point-of-care tests or throat culture. Where antibiotic therapy is indicated, most guidelines recommend narrow-spectrum β-lactams (penicillin-V or -G, amoxicillin, benzathine penicillin G), with first-generation cephalosporins or macrolides recommended for individuals with a penicillin allergy.^120^^,^^125^ We report that 59% of prescribed antibiotics were recommended for Strep A sore throat according to country-specific guidelines. Penicillin-based antibiotics, penicillin V and amoxicillin, were the most common antibiotic class prescribed globally, both of which were guideline-recommended in many of the countries included in this review. Penicillin V and amoxicillin are inexpensive, well-tolerated, and are universally effective against Strep A.^57^^,^^126^ First generation cephalosporins, also effective in the treatment of Strep A sore throat, are recommended for patients with penicillin allergy. However, despite not being recommended for Strep throat, broad spectrum second and third generation cephalosporins were used at similar rates.^127^^,^^128^ Amoxicillin-clavulanate (J01CR02) was the most prescribed broad-spectrum antibiotic across all countries, despite only being recommended for Strep A sore throat in six of the 35 studies reporting its use. Amoxicillin-clavulanate is rarely recommended for treatment of Strep A sore throat as the addition of clavulanate has shown to add no therapeutic advantage in clinical trials and is associated with increased adverse side effects and drug resistance.^129^^,^^130^

There are several limitations to consider in our analyses. Most studies included for analysis reported prescribing rates rather than dispensing or consumption rates which have been shown to be lower in some studies.^36^^,^^131^ In contrast, a meta-analysis of 38 studies from 24 countries reported that antibiotics are commonly dispensed without prescription.^132^ Whilst we did not intentionally exclude studies that reported antibiotics obtained over the counter, we were unable to find studies that provided both the number of antibiotics dispensed and a corresponding denominator to enable dispensing rate calculations. Further studies with appropriate study designs are required to better understand and quantify actual antibiotic consumption for sore throat.

Our study was also limited as we did not have all the data needed to accurately measure adherence to treatment guidelines. We referred to current antibiotic prescribing guidelines, which may differ from the prevailing advice at the time of the individual studies. We were also unable to differentiate what proportion of antibiotics were prescribed for patients with a penicillin allergy, as second-line therapy, or for children who may have concomitant infections; making this differentiation would be necessary to accurately report on treatment guideline adherence. For example the antibiotic prescription profile often differs between first episodes of sore throat and recurrent episodes.^133^ Whilst Strep A is universally susceptible to narrow-spectrum β-lactams by standard *in vitro* testing methods,^134^ a substantial proportion of cases (usually 10–20%)^135^ treated with β-lactams will suffer relapses. The relapse rate with β-lactams is higher than for broader-spectrum antibiotics,^136^^,^^137^ which likely contributes to clinical decisions to treat Strep A pharyngitis outside of guideline recommendations. Whilst it is expected that patients experiencing repeated episodes of sore throat would represent a small proportion of all cases, it is likely that at least some proportion of broad-spectrum antibiotics were prescribed in response to Strep throat recurrence, following ineffective prior treatment with narrow-spectrum antibiotics. The frequent use of non-recommended antibiotics and second-line antibiotics for patients with sore throat as observed in this study adds substantially to health care costs and promotes bacterial resistance.^138^

We reported on rates from the most recent years available to provide contemporary data on antibiotic prescription rates. This may aid other extensive international efforts (e.g., by US Centers for Disease Control and Prevention and others) to reduce inappropriate antibiotic prescribing. Large-scale studies reviewing rates of antibiotic prescribing over time would be useful to evaluate the impact of antimicrobial stewardship programs globally. Studies from the US reported that the antibiotic prescribing decreased from 76% of sore throat visits among adults (in 1989–1992)^61^ to 60% in 2000, after which it has remained relatively stable.^57^ The authors noted that, whilst the rate has stabilized, the prescription rate still far exceeds the 10% prevalence of Strep A among adults accessing health care for sore throat. Our meta-analysis from HICs found that at least 50% of prescriptions were for Strep A-positive patients. Intervention studies that aim to reduce antibiotic prescribing for sore throat by targeting policy, prevention, the prescriber, pharmacy, and patients have shown promising results. For example, the Global Respiratory Infection Partnership reported a 50% reduction in antibiotic prescribing following program implementation. Such results add plausibility to our upper bound estimates of courses averted in Scenario 2, which combines the potential effect of improved antibiotic stewardship and reduction in infection rate.

Overall, our findings indicated that there is little empirical data to estimate the global consumption of antibiotics due to sore throat, nor the proportion attributable to Strep A infection. The available data comes largely from HICs with electronic medical records that facilitate a linkage or connection between diagnosis or symptoms and treatment. Such data infrastructure is less prevalent in LMICs, so traditional community-based surveys may be required to better understand consumption rates and practices. These data are crucial to understanding the broader impact of Strep A vaccination—impact beyond a direct reduction in infections–and, therefore, its economic and societal value.

## Contributors

KM and JWC conducted the literature review and analyses and prepared the first draft manuscript. JWC conceptualised the study, and KM, TB, DC, DB, and JRC provided constructive feedback and suggestions to the study design. All authors contributed to reviewing and editing the final manuscript.

## Data sharing statement

The data that support the findings of this study are within the Supplementary Material and cited publications.

## Declaration of interests

The authors have no conflicts of interest to disclose.
