# Supplementary material for: Antibiotic consumption for sore throat and the potential effect of a vaccine against group A Streptococcus: a systematic review and modelling study
Source: eBioMedicine. 2023 Nov 9;98:104864. doi: 10.1016/j.ebiom.2023.104864 (PMC10663680; doi:10.1016/j.ebiom.2023.104864)
Supplement: Supplementary Material [file mmc1.docx]

**Supplementary Material**

**Calculations for the number of prescriptions to treat sore throat averted due to Strep A vaccination**

Scenario 1: No change in prescribing practices, but the proportion of prescriptions for sore throat that are attributable to Strep A are averted by vaccination.

We calculated the total number of prescribed courses averted for Scenario 1 as

$$S_{1}=\sum_{i} \left( n_{i}^{a}.r_{i}^{a} \right).p\left( StrepA \right).VE.VC$$

Where

$\sum_{i} (n_{i}^{a}.r_{i}^{a})$ is the sum of the population size *n* for age group *a* in country *i* multiplied by the observed prescribing rate *r* among age group *a* in country *i.*

p(StrepA) is the proportion of prescriptions attributable to Strep A

VE is vaccine efficacy

VC is vaccine coverage

Scenario 2: A change in prescribing practices among children presenting with sore throat in high-income countries in conjunction with a decrease in Strep A infection rates for all countries.

We calculated the total number of courses averted for Scenario 2 as

$$S_{2}=\left\{ \begin{aligned} \left( \sum_{i} \left( n_{i}^{a}.r_{i}^{a} \right)- \sum_{i} \left( n_{i}^{a}.m^{a} \right) \right)-\left( \sum_{i} \left( n_{i}^{a}.m^{a} \right).p\left( StrepA \right).VE.VC \right), &i=\text{high-income} \\ \sum_{i} \left( n_{i}^{a}.r_{i}^{a} \right).p\left( StrepA \right).VE.VC, &i=\text{low- and middle-income} \end{aligned} \right.$$

where

$m^{a}$ is the minimum observed prescribing rate for age group *a*

In the base case analysis,

$r_{i}^{a}$ = the prescribing rates for children and young adults summarised in Supplementary Table S3

$n_{i}^{a}$ = the population counts for 5-14 year-olds for each country with prescribing rates

$m^{a}$ = 0.014 (the prescribing rate among children and young adults observed in the Netherlands)

*p(StrepA)* = 0.5 (from our meta-analysis)

VE = 0.80 (WHO’s Preferred Product Characteristics for Strep A vaccines)

VC = 0.80 (assumed)

In the sensitivity analyses, we calculated averted prescriptions for a VE of 70%, 80%, and 90%; VC of 70%, 80%, and 90%; and duration of protection of 5 and 10 years, equal to prescriptions averted among populations, $n_{i}^{a},$ of 5-9 year-olds and 5-14 year olds respectively.
