# Supplementary material for: Antibiotic consumption for sore throat and the potential effect of a vaccine against group A Streptococcus: a systematic review and modelling study
Source: eBioMedicine. 2023 Nov 9;98:104864. doi: 10.1016/j.ebiom.2023.104864 (PMC10663680; doi:10.1016/j.ebiom.2023.104864)
Supplement: Supplementary Figs. S1–S3 and Tables S1–S7 [file mmc2.docx]

Supplementary Table S1: Numbers of studies included in each analysis stratified by country (references in superscript)

| **Country** |  | **Number of studies (reference(s))** | | |
| --- | --- | --- | --- | --- |
|  | **Total no. of unique studies** | **Prescribing rate** | **Prescribing attributable to Strep A** | **Antibiotic class** |
| Australia | 3 | 1^(38)^ | 1^(71)^ | 1^(104)^ |
| Bahrain | 1 |  |  | 1^(94)^ |
| Belgium | 1 | 1^(43)^ |  |  |
| Bosnia and Herzegovina | 1 |  |  | 1^(102)^ |
| Brazil | 1 |  |  | 1^(101)^ |
| Canada | 1 | 1^(53)^ |  |  |
| Croatia | 2 | 2^(34,45)^ |  | 2^(34,45)^ |
| Cyprus | 1 |  |  | 1^(89)^ |
| Denmark | 3 | 1^(20)^ | 1^(77)^ | 3^(20,77,116)^ |
| Finland | 1 |  |  | 1^(88)^ |
| France | 2 |  | 1^(68)^ | 1^(97)^ |
| Greece | 2 |  | 1^(76)^ | 1^(87)^ |
| India | 1 |  |  | 1^(86)^ |
| Indonesia | 1 |  |  | 1^(139)^ |
| Ireland | 1 |  |  | 1^(112)^ |
| Israel | 4 | 1^(36)^ | 1^(78)^ | 2^(93,109)^ |
| Italy | 3 | 2^(46,58)^ |  | 3^(46,58,82)^ |
| Japan | 2 | 1^(31)^ |  | 2^(31,117)^ |
| Latvia | 1 |  |  | 1^(105)^ |
| Lithuania | 1 | 1^(50)^ |  | 1^(50)^ |
| Malta | 1 |  |  | 1^(115)^ |
| Netherlands | 7 | 6^(24,33,43,44,49,56)^ |  | 2^(24,113)^ |
| Norway | 1 |  |  | 1^(84)^ |
| Pakistan | 2 |  |  | 2^(96,140)^ |
| Poland | 3 | 1^(62)^ | 1^(67)^ | 2^(62,103)^ |
| Portugal | 1 |  |  | 1^(79)^ |
| Russia | 1 |  |  | 1^(99)^ |
| Serbia | 1 | 1^(47)^ |  |  |
| Spain | 7 | 1^(37)^ | 2^(73,74)^ | 5^(37,81,90,91,110)^ |
| Sweden | 8 | 5^(23,26,40,42,43)^ | 3^(26,40,63)^ | 4^(40,63,98,106)^ |
| Switzerland | 1 |  |  | 1^(107)^ |
| Taiwan | 1 | 1^(48)^ |  |  |
| Thailand | 2 | 1^(28)^ |  | 2^(28,80)^ |
| Turkey | 3 |  |  | 3^(95,111,114)^ |
| UK | 10 | 7^(21,22,25,29,30,32,41)^ |  | 4^(25,92,100,108)^ |
| US | 20 | 11^(5,27,35,39,51,52,54,57,59-61)^ | 7^(64-66,69,70,72,75)^ | 8^(57,59,61,69,70,72,83,85)^ |
| Zambia | 1 | 1^(55)^ |  |  |
| **Total** | **103*** | **46*** | **18** | **62** |

*Includes one study reporting data for three countries.

Supplementary Table S2: Antibiotic prescribing rates for sore throat stratified by study country; all ages^()^

| Country | Reference | Period | Case definition (as defined by study authors) | Region | Clinical Setting (data source) | Age group | Rate (courses /100py) |
| --- | --- | --- | --- | --- | --- | --- | --- |
| US | Lewnard, 2020*^(35)^ | 2012-2015 | Strep throat and scarlet fever; pharyngitis; tonsillitis | National | GP (NAMCS); ED & OPD (NHMACS); retail clinics (MarketScan) | <65y (stratified) | 4·8 |
|  | Fleming-Dutra, 2016^(5)^ | 2010-2011 | Strep throat and scarlet fever; pharyngitis; tonsillitis | National | GP (NAMCS); ED & OPD (NHMACS) | All (stratified) | 4·3 |
|  | Mehrotra, 2015^(39)^ | 2007-2009 | Strep throat and scarlet fever; pharyngitis and laryngitis | National | GP (NAMCS); ED & OPD (NHMACS); retail clinics (MinuteClinic, TakeCare, LittleClinic) | >1y | 3·5 |
|  | Gonzales, 2001^(27)^ | 1998 | Strep throat and scarlet fever; pharyngitis; tonsillitis | National | GP (NAMCS) | All | 3·2 |
| UK | Gulliford, 2019^(29)^ | 2016 | Sore throat | National | GP (CPRD, 38 GPs) | All | 2·6 |
|  | Dolk, 2018^(25)^ | 2013-2015 | Sore throat | National (England) | GP (THIN, 349 GPs) | All | 3·4 |
|  | Hawker, 2014*ǂ^(32)^ | 2011 | Sore throat | National | GP (THIN, 537 GPs) | All | 3·0 |
|  | Gulliford, 2009ǂ^(30)^ | 2006 | Sore throat and pharyngitis; tonsillitis | National | GP (GPRD, sample from 78 GPs) | All | 5·5 |
|  | Smith, 2006ǂ^(41)^ | 2001 | Sore throat | National | GP (GPRD, 350 GPs) | All | 6·9 |
|  | Ashworth, 2006ǂ^(21)^ | 2000 | Sore throat | National | GP (GPRD, 108 GPs) | 1-84y (stratified) | 4·3** |
|  | Ashworth, 2004ǂ^(22)^ | 2000 | Sore throat; tonsillitis | National | GP (GPRD, 108 GPs) | All | 4·7** |
| Sweden | Tyrstrup, 2017^(43)^ | 2012 | Tonsillitis | Jonkoping County | GP (EMR) | ≥1y | 2·4 |
|  | Tyrstrup, 2016*ǂ^(42)^ | 2013 | Sore throat (inc tonsillitis, pharyngitis and mononucleosis, quinsy and scarlet fever) | National | GP (PRIS, 88 GPS) | All | 1·8 |
|  | Cars, 2017ǂ^(23)^ | 2015 | Tonsillitis | Stockholm County | GP and ED (VAL) | All | 2·4 |
|  | Neumark, 2010ǂ^(40)^ | 2005 | Pharyngitis; tonsillitis | Kalmar County | GP (Swedestar) | All | 1·9 |
|  | Engstrom, 2004^(26)^ | 2001 | Pharyngitis; tonsillitis | Ostergotlan, Kalmar and Jonkoping counties | GP (Swedestar, 11 GPs; BMS, 1 GP) | All | 2·9 |
| Netherlands | Hek, 2020*^(33)^ | 2014 | Throat symptom/complaint; tonsils symptom/complaint; strep throat; tonsillitis | National | GP (NPCD, 307 GPs) | All | 0·7 |
|  | Debets, 2017^(24)^ | 2012 | Tonsillitis | Utrecht | GP (JHN, 45 GPs) | All | 0·9 |
|  | Tyrstrup, 2017^(43)^ | 2012 | Tonsillitis | Utrecht | GP (JHN, 45 GPs) | ≥1y | 0·8 |
|  | van den Broek d’Obrenan, 2014ǂ^(44)^ | 2010 | Tonsillitis | Utrecht | GP (JHN, 45 GPs) | All | 1·7 |
| Australia | McCullough, 2017^(38)^ | 2010-2015 | Strep throat; tonsillitis | National | GP (Bettering the Evaluation and Care of Health study) | All | 3·2 |
| Belgium | Tyrstrup, 2017^(43)^ | 2012 | Tonsillitis | Flanders | GP (Intego Network, 51 GPs) | ≥1y | 1·6 |
| Croatia | Vojvodic, 2010*^(45)^ | 2003-2005 | Pharyngitis; tonsillitis | Eastern Croatia | GP (EMR, 30 practitioners) | All | 18·9 |
|  | Katic, 2000^(34)^ | 1994/95 | Pharyngitis | Zagreb | GP (EMR, 11 practitioners) | All | 4·4 |
| Denmark | Aabenhus, 2017^(20)^ | 2012/13 | Tonsillitis | National | GP (Danish National Prescription Database) | All | 1·7 |
| Israel | Low, 2018^(36)^ | 2015 | Strep throat; pharyngitis; tonsillitis | National | GP (Clalit Health Services) | All (stratified) | 7·8 |
| Japan | Hashimoto, 2020^(31)^ | 2012/13-2014/15 | Pharyngitis; tonsillitis; scarlet fever | National | Outpatient (National Database of Health Insurance Claims and Specific Health Checkups) | All (stratified) | 10·4 |
| Spain | Malo, 2015^(37)^ | 2009/2010 | Tonsillitis | Aragon | GP (EMR, 959 practitioners) | All | 2·4 |
| Thailand | Greer, 2018^(28)^ | 2015-2016 | Pharyngitis | Mueang Chiang Rai District | GP (EMR, 32 GPs) | All | 3·6 |

*Study used to represent country-level prescribing rates; **Age and sex standardized to European Standard Population

ǂ Study reported rates for multiple periods, most recent shown

Abbreviations: Y, year; py, person-year; GP, general practice; ED, emergency department; OPD, outpatient department. NAMCS, National Ambulatory Medical Care Survey; NHAMCS, National Hospital Ambulatory Medical Care Survey; C(G)PRD, Clinical (General) Practice Research Datalink; THIN, The Health Improvement Network; EMR, Electronic Medical Records; PRIS, Primary care Record of Infections in Sweden; VAL (Vårdanalysdatabasen), Stockholm regional health care data warehouse; NPCD, Nivel Primary Care Database; JHN, Julius General Practitioners’ Network.

Supplementary Table S3: Antibiotic prescribing rates for sore throat stratified by study country; children and young adults

| Country | Reference | Period | Case definition (as defined by study authors) | Region | Clinical Setting (data source) | Age group | Rate (courses /100py) |
| --- | --- | --- | --- | --- | --- | --- | --- |
| US | Lewnard, 2020*^(35)^ | 2012-2015 | Strep throat and scarlet fever; pharyngitis; tonsillitis | National | GP (NAMCS); ED & OPD (NHMACS); retail clinics (MarketScan) | <20y | 8·7 |
|  | Fleming-Dutra, 2016^(5)^ | 2010-2011 | Strep throat and scarlet fever; pharyngitis; tonsillitis | National | GP (NAMCS); ED & OPD (NHMACS) | <20y | 9·1 |
|  | Kronman, 2014^(51)^ | 2000-2010 | Strep throat and scarlet fever; pharyngitis; tonsillitis; pharyngoconjunctival fever | National | GP (NAMCS) | <18y | 8·1 |
|  | Linder, 2005^(52)^ | 1995-2003 | Sore throat (reason for visit) | National | GP (NAMCS); ED & OPD (NHMACS) | 3-17y | 6·5 |
|  | McCraig, 2002ǂ^(54)^ | 1999-2000 | Strep throat; pharyngitis; tonsillitis | National | GP (NAMCS) | <15y | 9·6 |
| UK | Ashworth, 2006ǂ^(21)^ | 2000 | Sore throat | National | GP (GPRD, 108 GPs) | 1-16y | 6·1** |
| Netherlands | Ivanovska, 2018*^(49)^ | 2012 | Strep throat; tonsillitis | National | GP (NPCD, 101 GPs) | <18 | 1·4 |
|  | Otters, 2004ǂ^(56)^ | 2001 | Strep throat; tonsillitis | National | GP (NPCD, 90 GPs) | <18 | 2·0 |
| Canada | Marra, 2006^(53)^ | 1996-2003 | Strep throat and scarlet fever; pharyngitis; tonsillitis | British Columbia | GP (PharmaNet; Medical Services Plan) | <15y | 7·1 |
| Israel | Low, 2018^(36)^ | 2015 | Strep throat and scarlet fever; pharyngitis; tonsillitis | National | GP (Clalit Health Services) | <19y | 21·5 |
| Italy | Barbieri, 2019^(46)^ | 2010-2015 | Strep throat and scarlet fever, tonsillitis | 12 regions | GP (Pedianet, 125 practitioners) | <15y | 11·0 |
| Japan | Hashimoto, 2020^(31)^ | 2012/13-2014/15 | Pharyngitis; tonsillitis; scarlet fever | National | Outpatient (National Database of Health Insurance Claims and Specific Health Checkups) | <20y | 22·4 |
| Lithuania | Karinauske, 2019^(50)^ | 2012 | Pharyngitis; tonsillitis | National | Outpatient (National Health Insurance Fund) | <19y | 21·8 |
| Serbia | Bozic, 2015^(47)^ | 2011-2013 | Tonsillitis | National | GP (National Health Insurance Fund) | <19y | 34·9 |
| Taiwan | Chang, 2017^(48)^ | 2000-2009 | Strep throat; tonsillitis (pharyngitis excl.) | National | GP (National Health Insurance Research Database) | <18y | 6·5 |
| Zambia | Musuku, 2017^(55)^ | 2014/2015 | Sore throat | Lusaka | School (Parent interview, 47 schools) | 5-29y (98% <20y) | 6·2 |

*Study used to represent country-level prescribing rates; **Age and sex standardized to European Standard Population

ǂ Study reported rates for multiple periods, most recent shown.

Abbreviations: Y, years; py, person-year; GP, general practice; ED, emergency department; OPD, outpatient department.

Supplementary Table S4: Antibiotic prescribing rates for sore throat stratified by study country; adults

| Country | Reference | Period | Case definition (as defined by study authors) | Region | Clinical Setting (data source) | Age group | Rate (courses /100py) |
| --- | --- | --- | --- | --- | --- | --- | --- |
| US | Lewnard, 2020*^(35)^ | 2012-2015 | Strep throat and scarlet fever; pharyngitis; tonsillitis | National | GP (NAMCS); ED & OPD (NHMACS); retail clinics (MarketScan) | 20-64y | 3·0 |
|  | Fleming-Dutra, 2016^(5)^ | 2010-2011 | Strep throat and scarlet fever; pharyngitis; tonsillitis | National | GP (NAMCS); ED & OPD (NHMACS) | 20-64y | 2·9 |
|  | Barnett, 2015^(57)^ | 1997-2010 | Sore throat (reason for visit) | National | GP (NAMCS); ED & OPD (NHMACS) | ≥18y | 1·8 |
|  | Hong, 2011^(59)^ | 1996-2006 | Strep throat and scarlet fever; nasopharyngitis; pharyngitis; laryngopharyngitis | National | GP (NAMCS); OPD (NHMACS) | ≥18y | 2·1 |
|  | Linder, 2003^(60)^ | 1995-2000 | Strep throat and scarlet fever; pharyngitis; tonsillitis; strep infection | National | GP (NAMCS) | 18-60y | 2·2 |
|  | Linder, 2001^(61)^ | 1989-1999 | Sore throat (reason for visit) | National | GP (NAMCS) | ≥18y | 2·5 |
| UK | Ashworth, 2006ǂ^(21)^ | 2000 | Sore throat | National | GP (GPRD, 108 GPs) | 17-84y | 3·8** |
| Israel | Low, 2018^(36)^ | 2015 | Strep throat; pharyngitis; tonsillitis | National | GP (Clalit Health Services) | ≥19y | 7·9 |
| Italy | Bianco, 2018^(58)^ | 2015-2016 | Pharyngitis | Unnamed city, Sothern Italy | GP (EMR, 5 practitioners) | ≥19y | 5·6 |
| Japan | Hashimoto, 2020^(31)^ | 2012/13-2014/15 | Strep A pharyngitis; Strep A tonsillitis; pharyngitis; tonsillitis; scarlet fever | National | Outpatient (National Database of Health Insurance Claims and Specific Health Checkups) | ≥20y | 7·9 |
| Poland | Panasiuk, 2010^(62)^ | 2005/2006 | Pharyngitis; tonsillitis | Lublin region | GP (EMR, 47 GPs) | Adults | 10·5 |

*Study used to represent country-level prescribing rates; **Age and sex standardized to European Standard Population; ǂ Study reported rates for multiple periods, most recent shown

Abbreviations: Y, years; py, person-year; GP, general practice; ED, emergency department; OPD, outpatient department.

Supplementary Table S5: Point-of-care or laboratory diagnostic outcome among sore throat patients who were prescribed antibiotics

| **Country** | **Reference** | **Period** | **Case definition** | **Region** | **Study design** | **Age group** | **N. (%*) of prescriptions** | | |
| --- | --- | --- | --- | --- | --- | --- | --- | --- | --- |
|  |  |  |  |  |  |  | Strep A+ | Strep A- | No test |
| Australia | Gunnarsson, 2021^(71)^ | 2014/15 | Sore throat | North Queensland | Prospective observational | All | 84 (72%) | 33 (28%) | - |
| Denmark | Stuhr, 2019^(77)^ | 2017 | Sore throat | Northern Denmark | Prospective observational | All | 219 (62%) | 114 (32%) | 19 (5%) |
| France | Faure, 2009^(68)^ | 2005-06 | Tonsillitis | Côte d'Or | Prospective observational | All | 98 (59%) | 13 (8%) | 55 (33%) |
|  |  |  |  |  |  | ≥15y | 57 (59%) | 12 (12%) | 28 (29%) |
|  |  |  |  |  |  | <15y | 41 (59%) | 1 (1%) | 27 (39%) |
| Greece | Plachouras, 2014^(76)^ | 2010 | Patients with RADT | Peloponnese | Prospective observational | All | 63 (65%) | 34 (35%) | - |
| Israel | Urkin, 2013^(78)^ | 2006 | Sore throat; pharyngitis; tonsillitis | Unnamed district | Retrospective observational | <19y | 1842 (27%) | 1839 (27%) | 3220 (47%) |
| Poland | Bura, 2017^(67)^ | 2014/15 | Sore throat | Poznan | Prospective case-control; case arm (Centor Score >1) | 18-44y | 24 (41%) | 35 (59%) | - |
| Spain | Molero, 2020^(74)^ | 2015 | Pharyngitis | 8 regions | Prospective before-after; 6y follow-up after introduction of RADT | ≥16y | 171 (50%) | 56 (16%) | 116 (34%) |
|  | Llor, 2011^(73)^ | 2008 | Pharyngitis | Catalonia, Spain | Prospective RCT; control arm (RADT and Centor Score >0) | 14-60y | 59 (46%) | 69 (54%) | - |
| Sweden | Andre, 2002^(63)^ | 2000 | Pharyngitis; tonsillitis | 5 counties | Prospective observational | All | 431 (59%) | 75 (10%) | 226 (31%) |
|  | Engstrom, 2004^(26)^ | 2001 | Pharyngitis; tonsillitis | 3 counties | Retrospective observational | All | 1021 (34%) | 441 (15%) | 1517 (51%) |
|  | Neumark, 2010^(40)^ | 2005 | Pharyngitis; tonsillitis | Kalmar County | Retrospective observational | All | 11645 (43%) | 5208 (19%) | 10057 (37%) |
|  | Tyrstrup, 2016^(42)^ | 2013 | Sore throat | National | Retrospective observational | All | N/A (60%) | N/A (13%) | N/A (27%) |
| US | Atlas, 2005^(64)^ | 2002/03 | Pharyngitis | Boston. MA | Prospective observational | ≥18y | 36 (55%) | 30 (45%) | - |
|  | Benin, 2003^(65)^ | 2001/02 | Pharyngitis; tonsillitis; scarlet fever | New Haven, CT | Retrospective observational | 3-18y | 47 (52%) | 26 (29%) | 17 (19%) |
|  | Fierro, 2014^(69)^ | 2009 | Pharyngitis; tonsillitis, strep throat and scarlet fever | Southeast PA & south NJ | Retrospective observational | <19y | 12445 (70%) | 4572 (26%) | 841 (5%) |
|  | Frost, 2019^(70)^ | 2007-17 | Patients with RADT or throat culture | North-central WI | Retrospective observational; random sample of electronic records | <19y | 590 (53%) | 514 (47%) | - |
|  | Havers, 2018^(72)^ | 2013/14 & 2014/15 | Pharyngitis; tonsillitis; scarlet fever | Seattle, WA; Marshfield, WI; Ann Arbor & Detroit, MI; Pittsburgh, PA; and Temple, TX | Prospective observational | All | 232 (53%) | 168 (38%) | 40 (9%) |
|  | Brennan-Krohn, 2018^(66)^ | 2011/12 | Pharyngitis patients with RADT or throat culture | Boston, MA | Retrospective observational | 3-18y | 21 (72%) | 8 (28%) | - |
|  | Nakhoul, 2013^(75)^ | 2009-10 | Pharyngitis | Cleveland, OH | Retrospective observational | ≥18y | 3634 (26%) | 7475 (53%) | 2938 (21%) |

*****Percent (%) of all antibiotic prescriptions for sore throat

*Supplementary Table S6: Joanna Briggs Institute Critical appraisal checklist for studies reporting prevalence data*

| **Study and year** | **JBI Critical appraisal checklist for studies reporting prevalence data** | | | | | | | | | |
| --- | --- | --- | --- | --- | --- | --- | --- | --- | --- | --- |
|  | Appropriate sample frame | Appropriate sampling of participants | Adequate sample size | Study subjects and setting adequately described | Data analysis conducted with sufficient coverage of sample | Valid methods for identification of condition | Condition measured in standard, reliable way | Appropriate statistical analysis | Adequate response rate | Total score |
| Andre, 2002^(63)^ | + | + | ? | + | ? | + | + | ? | ? | 5 |
| Atlas, 2005^(64)^ | + | + | ? | + | ? | + | + | + | ? | 6 |
| Benin, 2003^(65)^ | + | + | ? | + | + | + | + | + | + | 8 |
| Brennan-Krohn, 2018^(66)^ | − | + | ? | + | + | + | − | + | + | 6 |
| Bura, 2017^(67)^ | ? | ? | ? | + | + | + | + | + | − | 5 |
| Engstrom, 2004^(26)^ | + | ? | ? | − | + | + | + | + | + | 6 |
| Faure, 2009^(68)^ | + | + | ? | − | ? | ? | − | + | ? | 3 |
| Fierro, 2014^(69)^ | + | + | ? | − | ? | + | + | + | + | 6 |
| Frost, 2019^(70)^ | + | + | + | − | + | + | + | + | + | 8 |
| Gunnarsson, 2021^(71)^ | + | + | + | + | ? | + | + | + | ? | 7 |
| Havers, 2018^(72)^ | + | + | ? | + | ? | + | + | + | ? | 6 |
| Llor, 2011^(73)^ | - | + | + | + | + | + | + | + | + | 8 |
| Molero, 2020^(74)^ | + | + | ? | − | ? | + | + | + | ? | 5 |
| Nakhoul, 2013^(75)^ | + | + | + | − | + | + | + | + | + | 8 |
| Neumark, 2010^(40)^ | + | + | + | + | + | + | + | + | + | 9 |
| Plachouras, 2014^(76)^ | - | ? | ? | - | - | + | + | + | - | 3 |
| Stuhr, 2019^(77)^ | + | + | ? | + | ? | ? | ? | + | ? | 4 |
| Urkin, 2013^(78)^ | + | + | + | + | + | − | − | + | + | 7 |
| The score of each risk of bias domain for the listed studies by their respective symbol, with low risk (plus sign), unclear risk or not applicable (question mark), or high risk (dash). | | | | | | | | | | |

*Supplementary Table S7: Distribution of antibiotic classes prescribed for sore throat averaged across the studies*

| Antibiotic Class (ATC code*) | Country income group | | | |
| --- | --- | --- | --- | --- |
|  | All | High income | Middle income | Low income |
| Tetracyclines (J01A) | 0·8% | 0·8% | 0·7% | 0·6% |
| Amphenicols (J01B) | 0·1% | 0·1% | <0·1% | <0·1% |
| Beta-Lactam Antibacterials, Penicillins (J01C) | 70·1% | 70·9% | 71·0% | 59·0% |
| Other Beta-Lactam Antibacterials (J01D) | 11·0% | 10·4% | 13·6% | 13·3% |
| Sulfonamides and Trimethoprim (J01E) | 1·3% | 0·7% | 4·2% | 3·5% |
| Macrolides, Lincosamides and Streptogramins (J01F) | 12·6% | 13·2% | 7·1% | 14·1% |
| Quinolone Antibacterials (J01M) | 2·1% | 1·8% | 0·1% | 9·4% |
| Other Antibacterials (J01X) | <0·1% | <0·1% | <0·1% | <0·1% |
| Other, not classified | 2·0% | 2·0% | 3·3% | <0·1% |

*ATC codes: Anatomical Therapeutic Chemical Classification System

Supplementary Figure S1. Random-effects meta-analyses for proportion of sore throat prescriptions attributable to Strep A stratified by study country


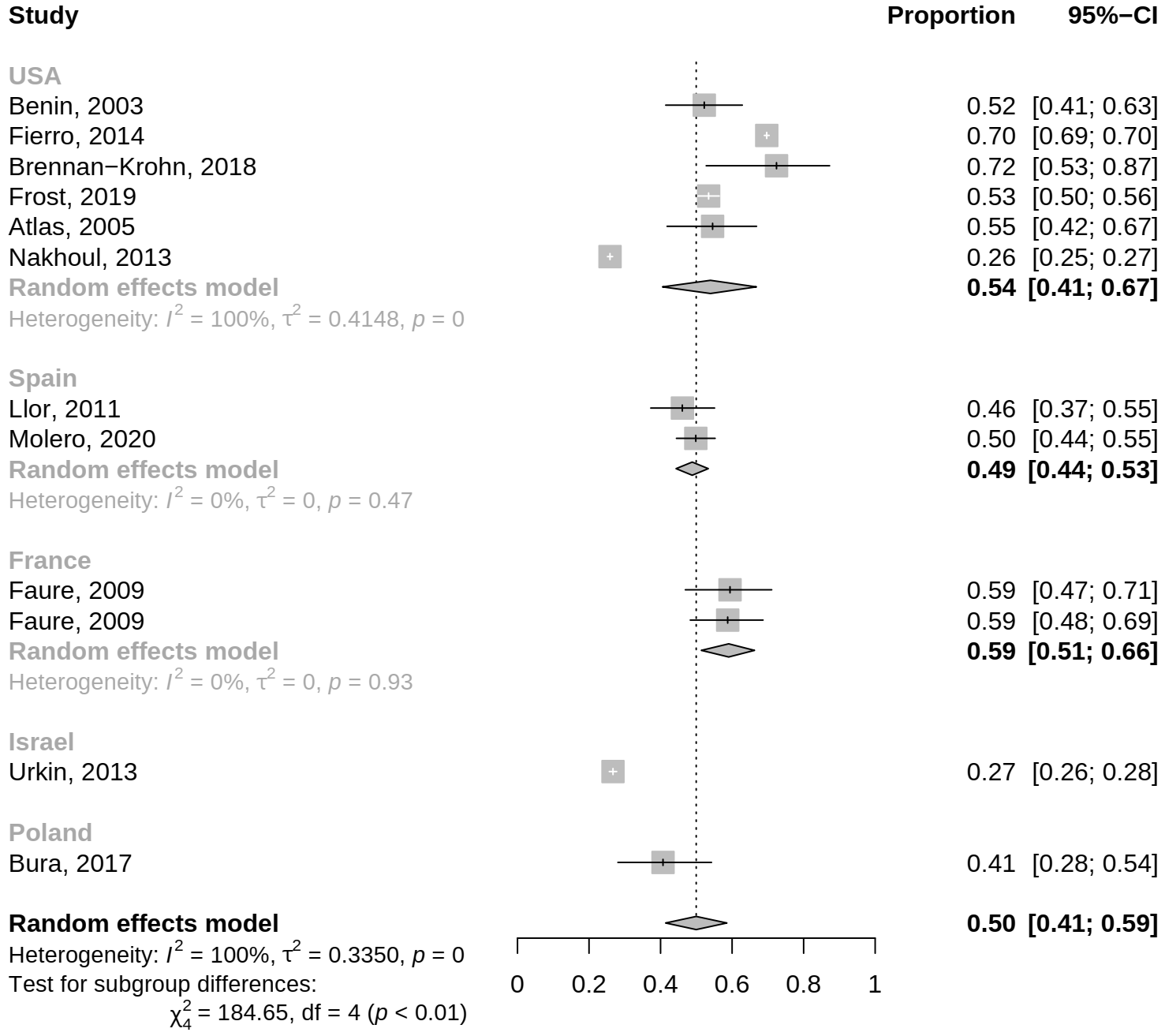


*Supplementary Figure S2. Sensitivity analysis for the reduction in antibiotic prescribing for sore throat due to Strep A vaccination (Scenario 2: A reduction in Strep A infections combined with a change in prescribing practices in HICs but not LMICs)*

*
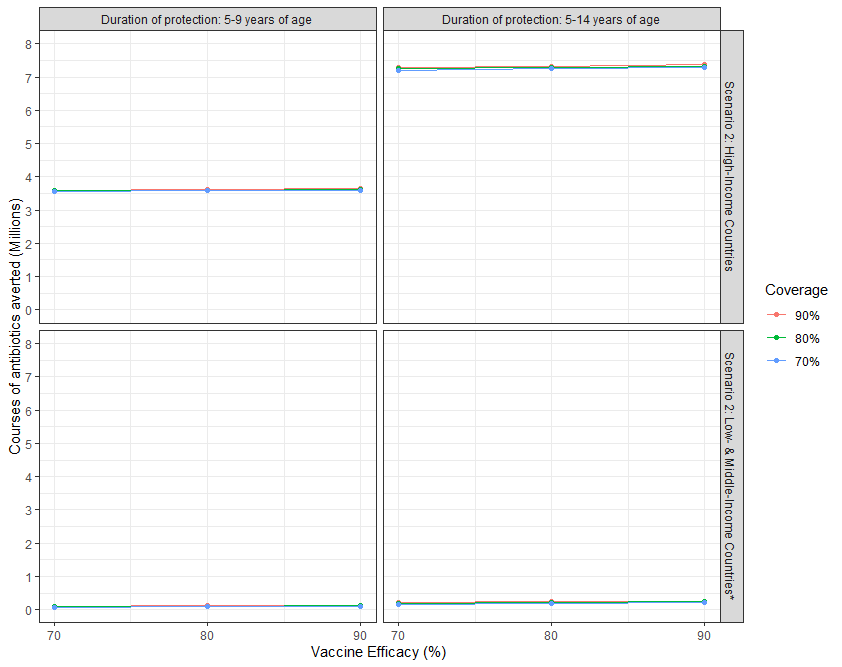
*

**Based on the population of two low- and middle-income countries.*

*Supplementary Figure S3. Distribution of antibiotic classes prescribed for sore throat stratified by study (first author) and country income level (top, high income countries; bottom-left, upper-middle income countries; bottom-right, lower-middle income countries).*


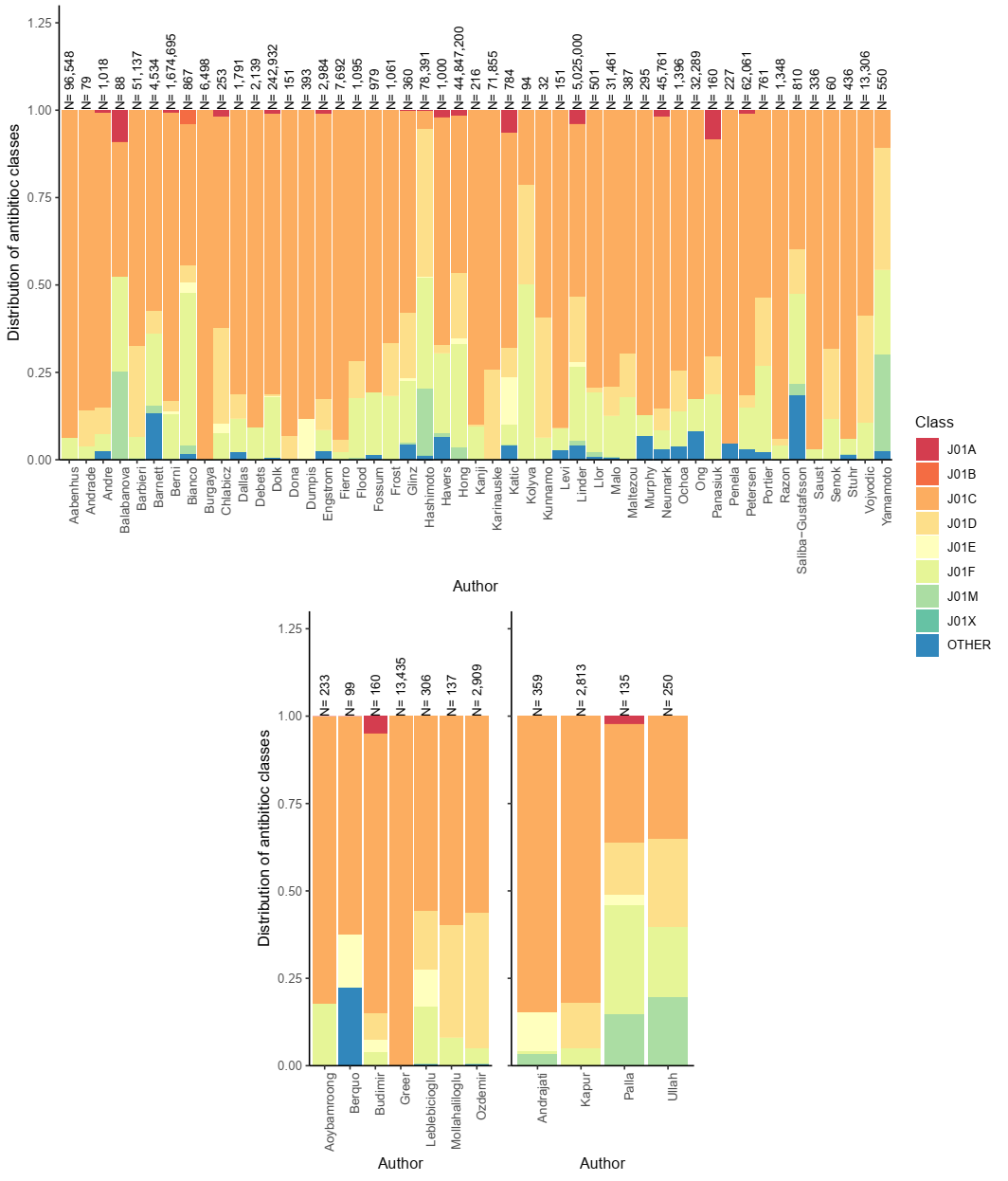


J01A Tetracyclines; J01B Amphenicols; J01C Beta-Lactam Antibacterials, Penicillins; J01D Other Beta-Lactam Antibacterials; J01E Sulfonamides And Trimethoprim; J01F Macrolides, Lincosamides And Streptogramins; J01M Quinolone Antibacterials; J01X Other Antibacterials; Other, not classified
